# A Phase I, open-label, dose escalation study of MGA271 in combination with pembrolizumab in patients with B7-H3-expressing melanoma, squamous cell cancer of the head and neck, or squamous cell non-small cell lung cancer

**DOI:** 10.1186/2051-1426-3-S2-P177

**Published:** 2015-11-04

**Authors:** Jan Baughman, Deryk Loo, Francine Chen, Paul Moore, Ezio Bonvini, James Vasselli, Jon Wigginton, Roger Cohen

**Affiliations:** 1 MacroGenics, San Francisco, CA, USA; 2 MacroGenics, Rockville, MD, USA; 3 University of Pennsylvania, Philadelphia, PA, USA

## Background

MGA271 is an Fc optimized humanized IgG1 monoclonal antibody that binds to B7-H3 (CD276), a member of the B7 family, currently undergoing Phase I testing. The Fc domain is engineered for enhanced binding to the activating FcγR, CD16A, and decreased binding to the inhibitory FcγR, CD32B. B7-H3 has limited expression in normal tissue and high expression in multiple tumors including melanoma (M), squamous cell cancer of the head and neck (SCCHN) and non-small cell lung cancer (NSCLC). The correlation between B7-H3 overexpression and poor prognosis in certain cancers suggests a role for B7-H3 in tumor escape. Despite the clinical success of agents including anti-CTLA-4, anti-PD-1 and anti-PD-L1 antibodies, the majority of patients with M, SCCHN or NSCLC progress nonetheless, and substantial unmet need exists for these patients. The underlying hypotheses for combining MGA-271 (anti-B7-H3) with pembrolizumab (anti-PD-1) are: 1) combining immune-modulating agents may mediate additive or synergistic antitumor activity (e.g. anti-CTLA-4+anti-PD-1), and can do so where neither single agent has pronounced antitumor activity (e.g. anti-PD-1+anti-LAG-3), 2) coordinate engagement of both innate and adaptive immunity, 3) both agents may enhance the immune response against tumors via modulation of T cell immunosuppression, and 4) limited expression of B7-H3 on normal tissues may help focus an immune attack on tumors, limiting the risk of immune-related adverse events (irAEs) resulting from the disruption of self-tolerance, allowing MGA271 to be combined more readily with other immune-modulating agents, including anti-CTLA-4 and anti-PD-1 antibodies. /PD-1 /PD-L1.

## Methods

This US, multi-center, open-label trial (NCT02475213) enrolls patients with advanced B7-H3-expressing SCCHN, M, or squamous NSCLC. Progression on previous checkpoint inhibitor is allowed. Following a 3+3 +3 dose escalation scheme, successive cohorts of patients will receive escalating doses of weekly IV MGA271 beginning at 3 mg/kg, and a fixed dose of IV pembrolizumab (2 mg/kg) administered every three weeks. Both study drugs will be administered starting on Day 1 of the study and given for up to one year. A three-cohort expansion phase will open at the established MTD with 16 patients each with M, SCCHN and NSCLC. The primary objective of this study is to determine the safety, dose-limiting toxicity and maximum tolerated dose of the MGA271/pembrolizumab combination. Secondary objectives include evaluation of pharmacokinetics, pharmacodynamics and preliminary anti-tumor activity of this combination. This novel study will provide the first clinical assessment of coordinated targeting of both the B7-H3 and PD-1/PD-L1 axes in patients with advanced cancer.

## Trial registration

ClinicalTrials.gov identifier NCT02475213.

